# Random variation of inspiratory lung function parameters in patients with COPD: a diagnostic accuracy study

**DOI:** 10.1186/1471-2466-10-28

**Published:** 2010-05-14

**Authors:** Frank J Visser, Sunil Ramlal, Ben Pelzer, PN Richard Dekhuijzen, Yvonne F Heijdra

**Affiliations:** 1 Dept. of Pulmonology, Canisius Wilhelmina Hospital, Nijmegen, 6532 SZ, the Netherlands; 2 Dept. of Pulmonology, Radboud University Medical Centre, Nijmegen, 6525 GA, the Netherlands; 3 Dept. of Social Science Research Methods, Radboud University, Nijmegen, 6525 GA, the Netherlands

## Abstract

**Background:**

In chronic obstructive pulmonary disease (COPD), the response of the forced expiratory volume in 1 second (FEV1) after bronchodilator application is weak. Inspiratory parameters like the forced inspiratory volume in 1 second (FIV1) and inspiratory capacity (IC) can be responsive to bronchodilators. In an individual patient with COPD, a significant bronchodilator response must at least exceed the random variation for that parameter. Therefore, it is important that the type of scatter is homoscedastic, as the chance of underestimating or overestimating the random variation for low or high parameter values is minimized. The aim of this study is to investigate the random variation (type and quantity) of inspiratory parameters.

**Methods:**

In 79 stable COPD patients, spirometry was performed.

The forced inspiratory volume in 1 second (FIV1), inspiratory capacity (IC), maximal inspiratory flow at 50% (MIF50) and peak inspiratory flow (PIF) were measured five times in one day and again within two weeks of the first measurement. The values of these parameters, taken within one hour, within one day and between two different days, were compared. The coefficient of repeatability (CR) was calculated, and, in addition, linear regression was performed to investigate the type of scatter (homo- or heteroscedastic) of the measured parameters.

**Results:**

The type of scatter was heteroscedastic for all of the parameters when the differences were expressed as absolute values; however, when the differences were expressed as the percent change from the initial values, we found a more homoscedastic scatter. The CR within one hour of each parameter expressed as the percent change from the initial value was: IC, 19%; FIV1, 14%; PIF, 18%; MEF50, 21%.

**Conclusions:**

To obtain a more homoscedastic scatter, percentage changes in FIV1, IC and MIF50 are more appropriate than absolute changes.

In an individual patient with COPD, a significant improvement for a particular parameter must at least exceed the above-mentioned CR.

## Background

The severity of chronic obstructive pulmonary disease (COPD) is defined by the degree of expiratory airflow limitation. It is essential for diagnosis and provides a useful description of the severity of pathological changes in COPD [1]. It is, however, well known that the correlation between the subjective improvements in dyspnea and the increases in Forced Expiratory Volume in 1 second (FEV1) after inhalation of bronchodilators is low [2-4].

Many COPD patients do not show significant reversibility of FEV1 after bronchodilators, as defined by a 12% improvement from the initial value and at least 200 ml [5], but may experience less dyspnea from their use. Taube and co-workers [4] demonstrated that this change in dyspnea may be related to improvements in inspiratory flow rates. These authors found that in patients with severe COPD (FEV1 mean was 38% of the predicted normal value), the reduction in dyspnea after the inhalation of a beta (2)-adrenoreceptor agonist was closely correlated to the change in parameters of forced inspiration, particularly for the forced inspiratory volume in 1 second (FIV1), but not with changes in parameters of forced expiration. They also concluded that, "In less severe COPD or asthma, the reduction in dyspnea was associated with the improvements in both FIV1 and FEV1, but in severe COPD with the improvement in FIV1 only" [6].

O'Donnell et al. found a correlation between the change of the Inspiratory Capacity (IC) after bronchodilator administration, dyspnea and duration of exercise [7,8]. In 2005, a published ATS/ERS statement on clinical pulmonary function testing [9] made no recommendations on the measurement of inspiratory parameters including FIV1. Therefore, it is unclear how FIV1 and other inspiratory parameters should be measured and which improvements in a patient are beyond random variation for these parameters after the use of bronchodilators or other interventions.

How FIV1 should be measured in patients with COPD was the subject of a previous study by our group. We found that the optimal FIV1 was obtained immediately after a slow expiration (in contrast to a forced expiration) and that at least five forced inspiratory maneuvers should be performed [10].

However, there is no clear consensus about how to express reversibility in subjects with airflow limitation [5]. The two most commonly described methods are the percent change of the initial value and the absolute change in the parameter value. As the percent change from the initial value is too sensitive at very low values, as measured in severe obstructive patients, a third method uses the percent change from the predicted normal value [5]. For the inspiratory parameters under study, no accepted predicted normal values are available; hence, we used the first two methods.

**Figure 1:**
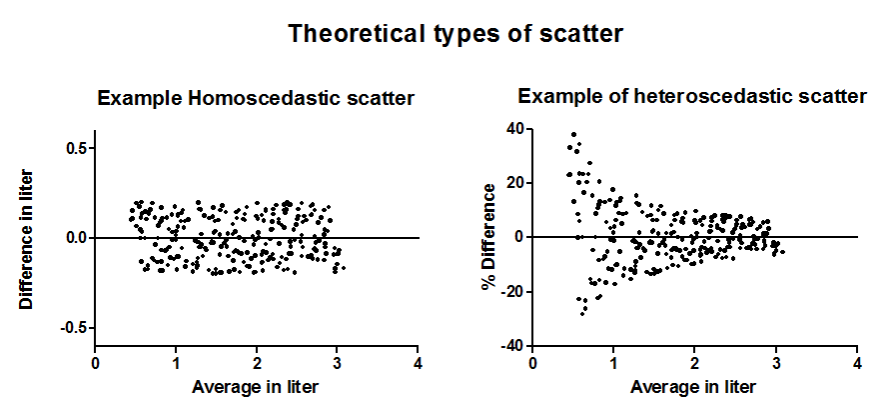
**Bland and Altman plots with theoretical types of scatter**. The vertical axis shows the test (T) retest (R) difference (T-R); the horizontal axis shows the average (T+R/2). Each point corresponds with one test-retest pair. Left panel: dataset with a random scatter not dependent on the parameter value. Right panel: same dataset, but now the percent change from the average parameter value is shown; the scatter is highly dependent on the parameter value. In the left panel, the scatter can be described with just one value, e.g., the coefficient of repeatability (CR). In the right panel, the scatter cannot be precisely described with one value because the scatter on the low and high parameter values will not precisely reflect a fixed CR. Thus, for describing random variation, a homoscedastic scatter is preferred.

For an individual patient with COPD, a significant bronchodilator response must at least exceed the random variation for the parameter of interest. Therefore, it is important to know which type of variation or scatter exists for that parameter. Figure 1 shows a theoretical dataset of a test-retest lung-function parameter with different types of scatter. In the left panel, we made the amount of scatter the same for each value of the parameter, called "homoscedastic" scatter. For the whole range of the parameter, we can use the same value for the random variation, and a difference of more than 0.2 is beyond the random variation. The same dataset is used in the right panel, in which the differences are related to the (average) parameter value (percent difference) but now the amount of scatter depends on the parameter value: the higher the value, the less (in this example) scatter or random variation there is; this type of scatter is called "heteroscedastic". Therefore, it is important to know the type of scatter of the parameters in which we are interested. A more homoscedastic scatter is desired when we express the differences as absolute differences or as relative to the parameter value.

The first topic of this study is to investigate which type of scatter applies to the (absolute and percent) changes in inspiratory parameters (FIV1, Inspiratory Capacity (IC), Peak Inspiratory Flow (PIF) and Maximal Inspiratory Flow at 50% (MIF50)). Next, we determine the coefficient of repeatability (CR) for the given parameters [5,11].

## Methods

A total of 79 (58 male) consecutive patients who met ATS-ERS [12] criteria for COPD were recruited from our outpatient clinic. Criteria for inclusion were a patient age ≥ 40 years, a smoker or former smoker (≥10 pack years), stable disease and an ability to perform lung function tests. Excluded patients were those on oral corticosteroids or antibiotics in the month before inclusion, or those who had symptomatic heart failure, respiratory diseases other than COPD, a history of asthma, allergic rhinitis or active cancer disease (except basal cell carcinoma of the skin). The study was approved by the Hospital Medical Ethical Committee, and all patients gave informed consent.

Patients were asked not to use short-term bronchodilators for the six to eight hours prior to the study and long-term bronchodilators for at least 12 hours before the study. Tiotropium and theophylline b.i.d. were not allowed to be used for the 24 hours prior to the spirometric test.

Before the tests, a 3.00-liter calibration syringe was used at three different emptying and filling speeds to check linearity, as recommended by ATS and ERS standards [9]. The ambient (room) temperature was measured before each test session so that BTPS corrections on the flows and volumes were adequately performed.

Lung function tests were performed five times on the first day (9, 10, 11, 14 and 15 hours) and once at nine hours within the following two weeks. Between the two days, the medication did not change. Also, on the second day the patients were requested to discontinue bronchodilators as on day one.

For expiratory parameters, three adequate and acceptable flow volume curves were produced in accordance with conventional ATS/ERS criteria [9]. The largest forced vital capacity (FVC) and FEV1 were recorded. For the predicted FEV1 and FVC, the normal values of the European Respiratory Society were used [13].

For inspiratory parameters, five adequate IC measurements and maximal forced inspirations after a slow and maximal expiration were obtained. Full inspiration was obtained when a plateau in the flow was reached or after at least an eight-second duration of the inspiration. Of these five maneuvers, we took the highest value obtained for the FIV1, IC, PIF and MIF50 [10]. IC was measured by the method described by Hadcroft and Calverly [14] immediately before each forced inhalation.

If, during the inspiratory maneuvers, the vital capacity (VC) was reached before the FIV1, then FIV1 = VC.

The flow-volume curves were measured with a V-MAX20 (Sensor Medics, ViaSys, Conshohocken, PA, USA).

In order to obtain proper inspiratory parameters after a slow expiration, we began the measurement during the slow expiration and stopped the procedure when the patient reached maximal inspiration; otherwise, the V-MAX20 software rejects the values obtained.

### Analysis

The five intra-day lung function parameter data were analyzed with the repeated measures ANOVA and Bonferroni's multiple comparison tests.

The type of scatter (homoscedastic or heteroscedastic) was determined as follows. The differences between each test and retest value pair versus the average value were plotted as described by Bland and Altman [11]. Negative differences were transformed to positive values by taking the absolute values of the differences. We applied linear regression of these transformed differences on the average value of the parameter. When there is a pure homoscedastic scatter, the regression line will be close to horizontal and the slope will not significantly differ from zero. When there is a heteroscedastic scatter, the slope of the regression line will be significantly different from zero. For each parameter, scatter plots were made for both absolute differences and percentage differences from the average value. With linear regression, we tested for the significances of the slopes. Instead of slopes, we present standardized slopes, i.e., correlation coefficients, as these can be compared across parameters and methods.

The coefficient of repeatability (CR), as established within one hour, intra-day and inter-day, was determined by the method described by Bland and Altman [11]. The CR was determined as 1.64 times the standard deviation of the differences, represented as absolute values or as percentages of the average values. We performed a one-tailed test instead of a two-tailed test, as the interventions we were interested in were expected to improve a parameter; hence, we took 1.64 times SD instead of 1.96 × SD. This use of a one-tailed test is analogous to the way in which lower limits of normals are calculated [13]. CR was used instead of the more common coefficient of variation (CV) because CV does not take into account the type of scatter that can be visualized by the scatter plots of Bland and Altman [11].

## Results

Seventy-nine patients were included for day one (intra-day measurements), and 76 were measured again within the following two weeks. For two patients, we were unable to get an appointment within the two weeks, and in one there was an exacerbation. The baseline patient characteristics are summarized in Table 1.

**Table 1:** Characteristics of the COPD patient group.

Number of subjects	79	Range
Male (%)	58 (73)	
Height, m, mean (SD)	1.702 (0.1)	1.49-1.94
Weight, kg, mean (SD)	75.4 (15.9)	39.7-128
BMI, kg/m^2^, mean (SD)	25.7 (4.96)	18-47
Age, yrs, mean (SD)	65.4 (8.69)	44-83
Current smoker (%)	47 (59)	
FEV1, mean (SD)	1.48 (0.70)	0.37-2.65
Predicted FEV1, mean (SD)	2.83 (0.60)	1,26-4.23
FEV1, % predicted, (SD)	48.7 (12.6)	13.5-79.8
GOLD 1 number (%)	13 (17)	
GOLD 2 number (%)	24 (30)	
GOLD 3 number (%)	28 (35)	
GOLD 4 number (%)	14 (18)	

COPD = Chronic Obstructive Pulmonary Disease; BMI = Body Mass Index; FEV1 = Forced Expiratory Flow in One second;

GOLD = Global Initiative for Chronic Obstructive Lung Disease (stage 1,2,3 or 4).

The intra-day mean and SD values did not differ significantly (repeated measures ANOVA) on different occasions that day (see Table 2). Therefore, we took three value pairs per parameter for each patient with a one-hour difference (9-10, 10-11 and 14-15 hours) for determination of the type of scatter and the one-hour coefficient of repeatability.

**Table 2:** Mean and (SD) values of lung function parameters on five occasions during the day

Parameter/time	9	10	11	14	15
FEV1	1.48 (0.70)	1.48 (0.69)	1.48 (0.90)	1.48 (0.70)	1.41 (0.68)
FIV1	2.70 (0.85)	2.71 (0.87)	2.69 (0.87)	2.65 (0.84)	2.62 (0.81)
IC	2.13 (0.69)	2.15 (0.68)	2.17 (0.72)	2.18 (0.75)	2.14 (0.79)
MIF50	4.68 (1.63)	4.61 (1.61)	4.65 (1.62)	4.51 (1.55)	4.47 (1.18)
PIF	4.96 (1.67)	4.92 (1.66)	4.95 (1.67)	4.89 (1.61)	4.84 (1.65)

FEV1 = Forced Expiratory Flow in one second; FIV1 = Forced Inspiratory Flow in One second; IC- Inspiratory Capacity; MIF50 = Maximal Inspiratory Flow at 50%; PIF = peak Inspiratory Flow.

### The type of scatter for the FEV1 and the inspiratory parameters FIV1, IC, PIF and MIF

**Figure 2:**
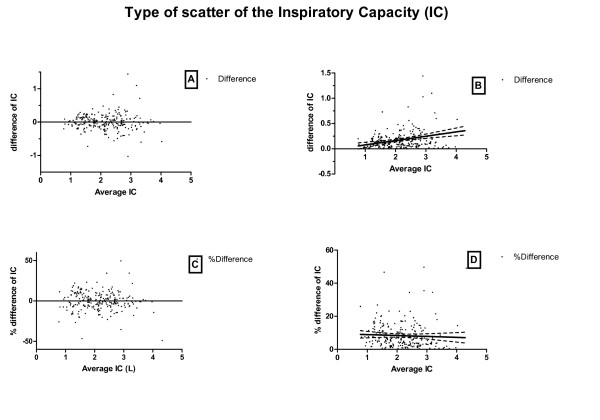
**Panel A: Bland-Altman plot showing the absolute difference of Inspiratory Capacity (IC) versus the average; it shows a heteroscedastic scatter of the IC**. Panel B: Absolute difference of IC versus average IC plus linear regression; this panel shows a slope in the regression line. Panel C: Difference IC now presented as the percentage of the average value; this panel shows a homoscedastic scatter. Panel D: Percentage difference of IC versus average IC plus linear regression; this panel shows a flat slope of the regression line, not significantly different from zero. The dashed lines in panel B and D represent the confidence interval of the regression line.

The scatter of differences in IC values versus average IC value on two occasions in-between one hour is shown in Figure 2, panel A. The scatter becomes wider when the average IC increases. Panel B presents the same data, except that negative difference values are made positive and a linear regression line is added to the figure. The regression line is not flat (P < 0.0001), as it would be if the scatter was independent of the IC (Table 3).

**Table 3:** Linear regression: standardized slopes (r) and P values; tests whether the slope is significantly different from zero. N = 237

Parameter	Standardized slope (r)	P value (significance from zero slope)
FEV1	0.37	<0.0001
%FEV1	0.10	0.1 (NS)
IC	0.27	<0.0001
%IC	0.05	0.46 (NS)
FIV1	0.20	0.002
%FIV1	0.13	0.045
PIF	0.20	0.002
%PIF	0.24	0.0002
MIF50	0.22	0.0009
%MIF50	0.14	0.0267

**Figure 3:**
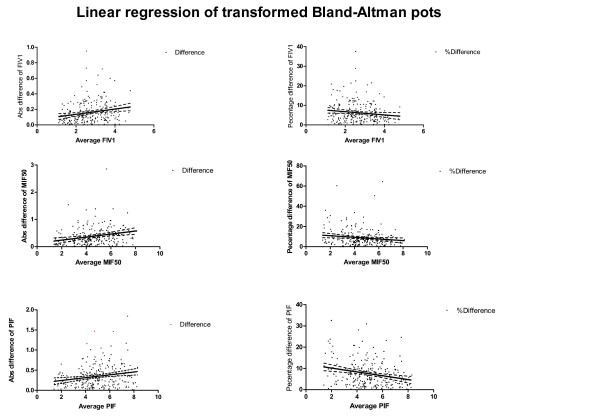
**Left panels show the scatter (absolute differences and regression lines for FIV1, MIF50 and PIF)**. Right panels show the percentage differences for the same parameters. The dashed lines represent the confidence intervals of the regression lines. All slopes of the regression lines are significantly deviated from flat (zero); however, apart from the PIF, the percentage differences show regression lines that are more flat (closer to a zero slope). FIV1 = Forced Inspiratory Flow in One second; MIF50 = Maximal Inspiratory Flow at 50%; PIF = peak Inspiratory Flow.

FEV1 = Forced Expiratory Flow in one second; FIV1 = Forced Inspiratory Flow in One second; IC- Inspiratory Capacity; MIF50 = Maximal Inspiratory Flow at 50%; PIF = peak Inspiratory Flow. See also Figures 2 and 3 and the text for further explanation.

On the other hand, when the difference in IC is expressed as a percentage of the average IC (Figure 2, panel C), an evenly distributed scatter can be seen along the whole range of the average IC. The slope of the linear regression line is now nearly flat (Figure 2, panel D) and is not significantly different from zero (Table 3). The other parameters were investigated in the same way as the IC, and the results of the linear regression are presented in Table 3. To visualize the (more homoscedastic) spread, the scatter plots of the FIV1, IC, MIF50 and PIF expressed as percentage differences can be seen in Figure 3.

We did not find a flat regression line for either presentation of the parameters (as differences in liters or as percentage differences of the average value); however, for all but the PIF, we found a more flat regression line corresponding to a lower (r) value when the percentage difference of the average value was used (less significant difference from zero, as can be seen in Table 3).

### The random variability presented by the coefficient of repeatability

**Figure 4:**
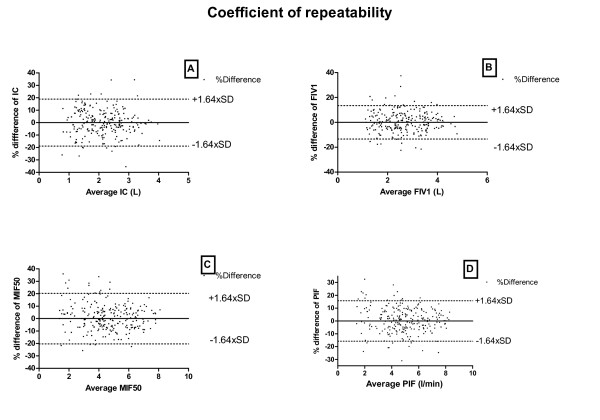
**Panels A-D show Bland-Altman plots of the percentage difference versus the average of the IC, FIV1, MIF50 and PIF**. The coefficient of repeatability (CR) corresponds with the dotted lines; in panel A, e.g., the coefficient of repeatability takes the value = 18.9%, which is 1.64xSD. IC- Inspiratory Capacity; FIV1 = Forced Inspiratory Flow in One second; MIF50 = Maximal Inspiratory Flow at 50%; PIF = peak Inspiratory Flow; SD = standard deviation.

The coefficients of repeatability for IC, FIV1, MIF50 and PIF are graphically presented as Bland-Altman plots in Figure 4, panels A-D. The spread around no difference (solid line) can be seen, and the coefficient of repeatability is presented as the dotted lines ±1.64 × standard deviation. We can also see the relatively even spread around the solid line, which is an indication of a more homoscedastic spread. In patients with COPD, we found that the one-hour random variabilities expressed as the coefficients of repeatability (CR) for the lung function parameters are: IC: 19%, FIV1: 14%, PIF: 18% and MEF50: 21% (Table 4).

**Table 4:** Coefficients of repeatability (CR) retested after one hour (n = 237), after five hours (n = 79) and after 3-10 days (n-76) (in % from initial value/absolute value)

Parameter	Retest after one hour	Retest after five hours	Retest another day
FEV1	11.8	13.7	17.9
IC	18.9	21.3	22.7
FIV1	13.5	17.9	18.0
PIF	17.9	17.8/0.90	17.9/0.85
MIF50	20.4	21.0	19.2

FEV1 = Forced Expiratory Flow in one second; FIV1 = Forced Inspiratory Flow in One second; IC- Inspiratory Capacity; MIF50 = Maximal Inspiratory Flow at 50%; PIF = peak Inspiratory Flow.

In the same way as the one-hour coefficients of repeatability, the intra-day coefficients of repeatability and the in-between day coefficients of repeatability are investigated.

The intra-day random variabilities expressed as the coefficients of repeatability (CR) for the lung function parameters are: IC, 21%; FIV1, 18%; PIF, 18% or 0.90 l/; and MEF50, 21% (Table 4).

The inter-day random variabilities expressed as the coefficients of repeatability (CR) for the lung function parameters are: IC: 23%, FIV1: 14%, PIF: 18% and MEF50: 21% (Table 4).

## Discussion

### Type of scatter

This study has shown that within the same subject, differences in lung function parameters (IC, FIV1, MIF50 and PIF) before and after one hour can more appropriately be described when taken as the percentages of the initial values than as differences in the absolute values because of the more homoscedastic scatter. All measured parameters showed a scatter that was significantly dependent on the average parameter value and thus is heteroscedastic if we present the differences in liters or L/sec. On the other hand, if we represent the difference as the percentage of the average value (which is nearby the initial value), we found no significant dependence on the parameter value for IC and less dependence on the values for the FIV1 and MIF50.

Several studies have addressed the variability of lung function parameters, especially on forced expiration [15-19], but used the variation coefficient instead of the method described by Bland and Altman [11]. Therefore, the type of spread was not investigated. In the ATS-ERS statement, the method of Bland and Altman is described as the preferred method for investigating the random variation, and this method makes the type of spread visible [5,11].

The only exception is the PIF, which displays a slightly steeper slope when expressed as the percentage difference.

### Random variability

The one-hour repeatability is by far the most important random variability because most interventions we are interested in, such as bronchodilator response, can be measured within one hour. Subjects must at least exceed this random variation before it can be decided that an improvement of an intervention can be attributed to that intervention. We did not find any CR for inspiratory parameters in the literature.

We decided to pool our patients' data (with one-hour differences measured at three time-points a day per patient) to obtain more data points and, thus, more reliable results. This pooling was possible because we found no significant differences between the group means and spreads and no significant differences of the parameters between the measurements between 9 a.m. and 2 p.m. This result is in contrast with Calverley et al. [20] and van Noord et al. [21], who found significantly lower values at 3 and 6 a.m.; however, we did not measure at these hours.

We chose the CR instead of the more popular variation coefficient because it more precisely reflects the repeatability and provides a graphical representation of the type of scatter, as stated by Bland and Altman [11] and the recommendations of the ERS-ATS committee [5].

The CR for the PIF is less than that for the MIF50 which may be because the MIF50 is situated near the PIF in maximal inspiratory flow volume curves but is seldom exactly aligned; thus, the MIF50 demonstrated more spread.

Whether PIF improvement is therefore more sensitive to bronchodilators than PIF is not answered by this study.

The intra-day coefficients of repeatability are important to know when we are performing interventions that take more than one hour, i.e., medications such as theophylline, tiotroprium or other interventions that take more time to retest.

We selected the 9-to-14-hour difference because all parameters as group means did not change during this interval. There was a small but significant decrease in some parameters (FEV1 and MIF50) on the 9-to-15-hour interval; therefore, we took the 9-to-14 as our difference. We think that this decrease in some parameter values may be due to the fact that patients at the end become tired of repeating this procedure five times a day, during which time they were not allowed to take any bronchodilator drugs, or that there may be some circadian effect [20,21]. The higher intraday CR value, than the one hour CR value, could be expected because of the greater time interval.

Improvements of interventions taking more than one day can be considered as beyond random variation when the inter-day coefficients of repeatability are taken into account. Between the two days, patients did not change their medication and no exacerbations occurred. A weak point of these CR is that we were unable to see all patients on an exact inter-day interval of one week because we were dependent on when our patients were able to visit our outpatient department again. As the smallest interval was three days, and the greatest interval was eight days, the analyses were all conducted within two weeks. In general, the longer the interval between the two measurements (from one hour to several days), the greater the CR obtained. This result may be caused by the longer time period for weather to influence the patients or other effects of irritants in the environment.

Similar to the one-hour and intra-day random variation, we were unable to find the inter-day random variation on inspiratory parameters in the literature.

Limitations of this study:

The subjects in this study include the investigation of only patients with COPD, so it does not extend to normal patients or those with asthma or restrictive disease. The random variation in these groups may be different.

The type of scatter was only examined after one hour, and it may be different when other intervals are taken into account.

The wash-out time for Tiotropium was 24 hours, although some investigators used 48 hours for this drug. We think this 24 hours time period had limited influence on the test-retest results.

We used all data including the outliers to construct the Bland and Altman plots; in small samples, this can influence the linear regression of the transformed Bland and Altman plots when the outliers are in the lower or upper zones of the average parameter value.

## Conclusions

Differences in lung function parameters (IC, FIV1, MIF50 and PIF) are described with less dependence on the parameter values when taken as percentages from the initial values than as absolute difference values.

The random variation expressed as coefficients of repeatability for several time intervals are presented.

## Competing interests

The authors declare that they have no competing interests.

## Authors' contributions

FV: initiated the study, supervised the lung function analyses and drafted the manuscript. SR: wrote the study protocol, corresponded with the medical ethical committee and collected the data. BP: advised about the method and statistics used, also edited the first revision of the manuscript. RD: participated in the design of the study. YH: participated in the design and edited the first versions of the manuscript. All authors read and approved the final manuscript.

## Pre-publication history

The pre-publication history for this paper can be accessed here:

http://www.biomedcentral.com/1471-2466/10/28/prepub
